# Using a Bayesian modelling approach (INLA-SPDE) to predict the occurrence of the Spinetail Devil Ray (*Mobular mobular*)

**DOI:** 10.1038/s41598-020-73879-3

**Published:** 2020-11-02

**Authors:** Nerea Lezama-Ochoa, Maria Grazia Pennino, Martin A. Hall, Jon Lopez, Hilario Murua

**Affiliations:** 1 AZTI-Tecnalia, Marine Research Division, Herrera Kaia, Portualdea z/g, 20110 Pasaia, Spain; 2 Inter-American Tropical Tuna Commission, Ecosystem and Bycatch Program, La Jolla, San Diego, CA USA; 3 Instituto Español de Oceanografía (IEO), Vigo, Spain; 4 International Seafood Sustainability Foundation (ISSF), Washington, DC USA

**Keywords:** Conservation biology, Ecological modelling, Marine biology

## Abstract

To protect the most vulnerable marine species it is essential to have an understanding of their spatiotemporal distributions. In recent decades, Bayesian statistics have been successfully used to quantify uncertainty surrounding identified areas of interest for bycatch species. However, conventional simulation-based approaches are often computationally intensive. To address this issue, in this study, an alternative Bayesian approach (Integrated Nested Laplace Approximation with Stochastic Partial Differential Equation, INLA-SPDE) is used to predict the occurrence of *Mobula mobular* species in the eastern Pacific Ocean (EPO). Specifically, a Generalized Additive Model is implemented to analyze data from the Inter-American Tropical Tuna Commission’s (IATTC) tropical tuna purse-seine fishery observer bycatch database (2005–2015). The INLA-SPDE approach had the potential to predict both the areas of importance in the EPO, that are already known for this species, and the more marginal hotspots, such as the Gulf of California and the Equatorial area which are not identified using other habitat models. Some drawbacks were identified with the INLA-SPDE database, including the difficulties of dealing with categorical variables and triangulating effectively to analyze spatial data. Despite these challenges, we conclude that INLA approach method is an useful complementary and/or alternative approach to traditional ones when modeling bycatch data to inform accurately management decisions.

## Introduction

The use of Species Distribution Models (SDMs) in conservation ecology has increased substantially in recent years. SDMs seek to link species presence/absence or abundance information with environmental variables to predict the probability of a species being found in non-sampled places or time periods^1^. SDMs have recently been used to identify and manage priority areas or “hotspots” of vulnerable species. Therefore, to protect these areas, it is essential that they are identified correctly. A variety of methodological approaches have been developed over the last decades to generate SDMs, such as Artificial Neural Networks (e.g., SPECIES), Classification and Regression Trees (e.g., BIOMOD), Maximum Entropy (e.g., MAXENT), Climatic Envelops (e.g., BIOCLIM), and regression models, such as Generalized Linear and Additive Models (GLM/GAM)^2–8^. However, the statistical challenges using SDMs have increased as datasets have become more complex over time^9^. Indeed, the need to account for spatial and temporal autocorrelations in data is now common when modelling complex non-linear relationships between species and the environment and quantifying the various sources of uncertainty associated with input data, sampling processes, observer biases and analytical errors^9^. If these issues are ignored in SDMs the models could generate misleading estimations of species-environment relationships and misidentifications of predicted suitability areas.

Within this context, Bayesian models are able to incorporate our knowledge of the unknown parameters of SDMs that govern species behavior, expressed through probability distributions, rather than just fixed estimates, as in frequentist approaches^10^. These resulting probability distributions are also the result of joining previous knowledge of the parameters with the observed data.

However, predicting the behavior of a species also requires knowledge of its spatial and temporal nature. Generally when geo-referenced species data are analyzed geographic coordinates (latitude and/or longitude) and temporal factors (e.g., year, month, etc.) are included in SDMs as continuous explanatory variables. Consequently, the spatial and temporal dependencies of observations are not taken into account. Hierarchical Bayesian models extend the concept of spatial and temporal autocorrelation in multilevel structures that include spatial and temporal random effects, and represent all the spatiotemporal variability that may have an effect on the species patterns^11^.

Nevertheless, as is the case in nearly all complex Bayesian models, posterior distributions and posterior predictive distributions attained from SDMs do not yield analytical expressions and, therefore, numerical methods are needed to approach them. In this sense, the most commonly used simulation-based approach is the Markov Chain Monte Carlo (MCMC) technique, despite it being computationally intensive^12,13^. By contrast, the Integrated-Nested Laplace Approximation (INLA) framework proposed by Rue, et al.^14^ is a relatively novel, and much faster alternative to MCMC.

Recently, researchers have been turning to INLA to model spatial and temporal fisheries data (e.g., trawler and gillnet fisheries)^15–22^, as they produce more realistic and accurate predictions than conventional models^31,33,37,40–43^. However, with regard to the tropical tuna purse-seine fisheries, INLA has only recently been explored for tuna and non-target species^23,24^ but has yet to be used for particular vulnerable bycatch species, such as sharks, turtles or mobulid rays.

Eastern Tropical Pacific tuna purse seine fisheries capture the greatest numbers of mobulids in bycatch compared to other gears and regions^25–27^. The Spinetail Devil Ray, or *Mobula mobular* (Müller & Henle, 1841), is one of the most frequently caught mobulid bycatch species in eastern tropical Pacific tuna purse-seine fisheries^27–29^. The taxonomy of the Genera *Manta* and *Mobula* have recently been revised^30^ and *Mobula japanica* has been included under *Mobula mobular*. The International Union for Conservation of Nature (IUCN) Red List of Threatened Species (https://www.iucnredlist.org/) lists it as “Endangered” globally. *Mobula mobular* is circumglobally distributed in tropical and subtropical waters, both in coastal and oceanic pelagic habitats^31,32^. Thus, accurately predicting hotspot areas (e.g., nurseries, reproductive, feeding, etc.) for this species is of vital importance to developing effective fishery management options.

This study aims to describe the use of the INLA-SPDE Bayesian approach by using Generalized Additive Models to predict the occurrence of *Mobula mobular* taken incidentally in the tropical tuna purse-seine fishery of the eastern Pacific Ocean using IATTC observer bycatch data. In doing so, this study initiates a discussion about the different models to obtain accurate spatial predictions of vulnerable bycatch species, such as *M. mobular*, for conservation and management purposes.

## Results

All the models that included the spatial effect showed lower DIC than those without it (Supplementary Table S4). Similarly, most of the models that do not account for non-linear relationship showed higher DIC values than the ones using smoothing functions. When the type of set was included as a dummy variable good prediction performance statistics and smoother predictions were obtained (Supplementary Table S4). Based on the combination of different aspects to obtain the most accurate model, both in terms of estimations and predictions (AUC, Sensitivity, Specificity, prediction and DIC values), the best fit INLA model included presence-absences as the response variable and oxygen, chlorophyll, nitrate, sea surface temperature, month and type of set as explanatory variables. The spatial effect was included in the model.

**Figure 1:**
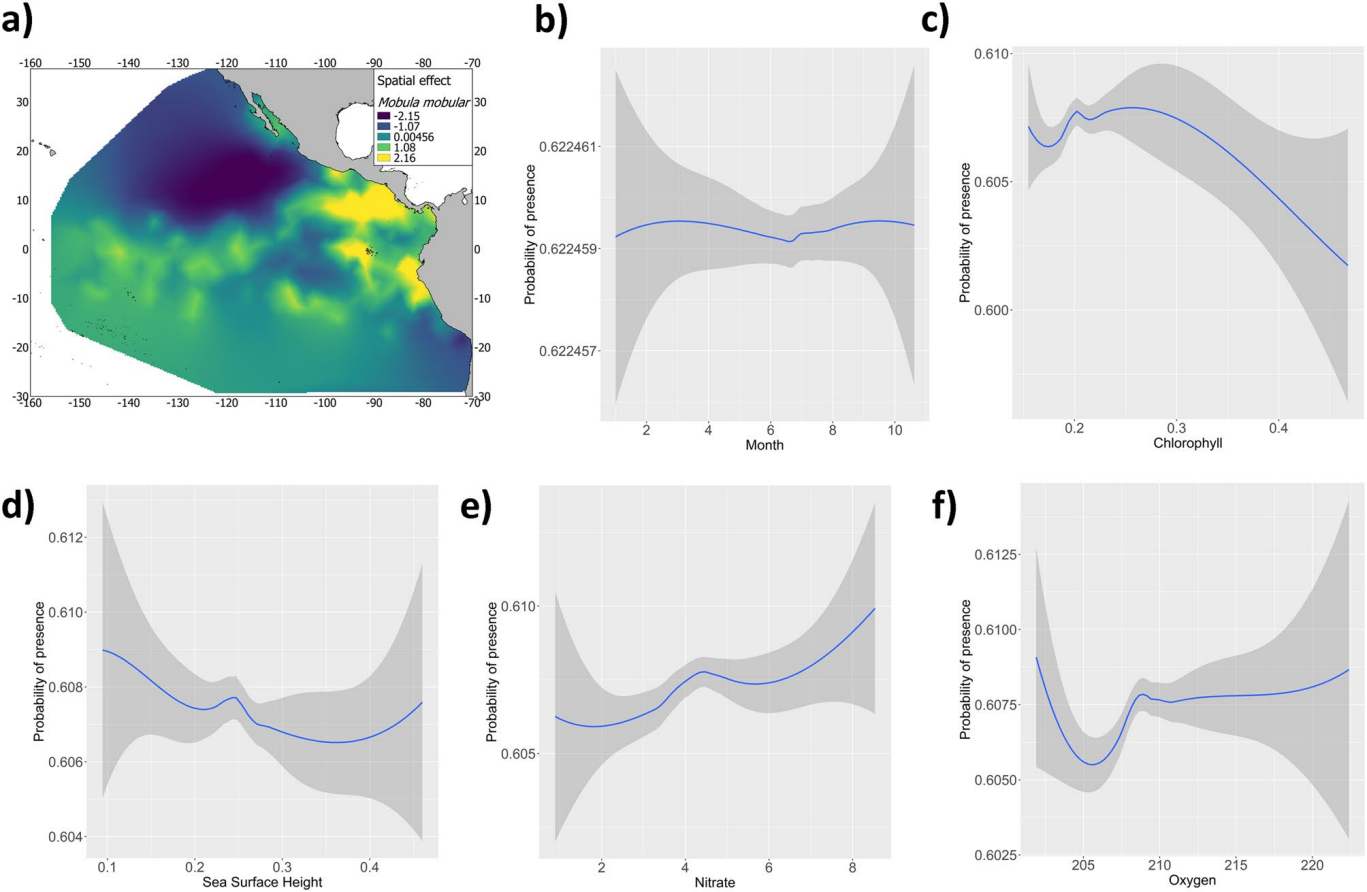
** (a)** The posterior mean of the spatial effect and the smoothed fits of covariates modeling the presence of *Mobula mobular* for: (**b)** Month, (**c)** Chl (chlorophyll, in mg·m^−3^ in x-axis), (**d)** SSH (sea surface height, in cm in x-axis), (**e)** Ni (nitrate, in mg/l in x-axis) and (**f)** O_2_ (oxygen, in mg/l in x-axis) variables. The y-axis represents the spline function. Shaded polygons indicate approximate 95% credible intervals bounds. Maps were created using Quantum GIS geographic information system. Open source geospatial foundation. URL: https://qgis.osgeo.org (2014).

The final INLA-SPDE (option 10) model had both the lowest DIC (8773.68) and LCPO (3.66), compared to the others (see Supplementary Table S4). The mean posterior probability of occurrence, the standard deviation and the first and third quartiles for each parameter of the fixed effects included in the final model are shown in Table 1. Results showed a positive relationship between chlorophyll and the presence of *M. mobular* between 0.1–0.2 mg·m^−3^. Similarly, results demonstrated that higher occurrences of *M. mobular* are expected to be found in waters with oxygen concentrations between 210–220 mg/l and low-medium nitrate concentrations (Fig. 1). A negative correlation was also identified between sea surface height values and the probability of occurrence of *M. mobular,* with higher probability in low SSH. Finally, the highest probability of presence of *M. mobular* was found mainly during winter (Fig. 1). The lowest relationship between the type of set and the presence of the species was found in Floating object sets (posterior mean = − 1.918; SD = 18.239); compared with the presence in School (posterior mean = 1.026; SD = 18.239) and Dolphin sets (posterior mean = 0.917; SD = 18.239) (Table 1).

**Table 1:** Numerical summary of the marginal posterior distribution of the fixed effects for the best INLA model for *Mobula mobular*.

Species	Predictor	Mean	SD	Q_0.025_	Q_0.5_	Q_0.975_
*Mobula mobular*	(Intercept)	0.000	31.406	− 61.746	0.005	61.656
	Dolphin set	0.917	18.239	− 34.895	0.917	36.697
	Floating object set	− 1.918	18.239	− 37.730	− 1.919	33.862
	School set	1.026	18.239	− 34.786	1.026	36.806

For each variable the mean, standard deviation, median (Q_0.5_) and a 95% credible intervals (Q_0.025_–Q_0.975_) are provided, containing 95% of the probability under the posterior distribution.

The overall predictability of the models was evaluated using the Area Under the receiver-operating Curve (AUC), Sensitivity, Specificity and Kappa. Kappa measures the proportion of correctly classified presence and absence after accounting for the probability of chance agreement^17^. AUC values were around 0.80–0.90, which indicates good model prediction performance and an excellent degree of discrimination between the locations with species presence and absence. All Kappa values were around 0.14, which indicates a good degree of similarity between the predicted species occurrence and the observations. Sensitivity (0.40–0.70) and Specificity (0.80–0.90) values were also good, which reflects the ability of the model to correctly predict true negative and true positive predictions (Table 2).

**Table 2:** Model prediction performance statistics for the 5 INLA interactions.

Interaction INLA	DIC	AUC	Kappa	Sensitivity	Specificity
1.0	8944.3	0.9	0.0	1.0	0.8
2.0	8879.2	0.9	0.4	0.4	0.9
3.0	8695.6	0.8	0.0	0.7	0.9
4.0	8688.7	1.0	0.3	0.4	1.0
5.0	8660.6	0.8	0.0	0.5	0.8

Statistics acronyms are: deviance information criterion (DIC), area under the curve (AUC), kappa, sensitivity and specificity.

**Figure 2:**
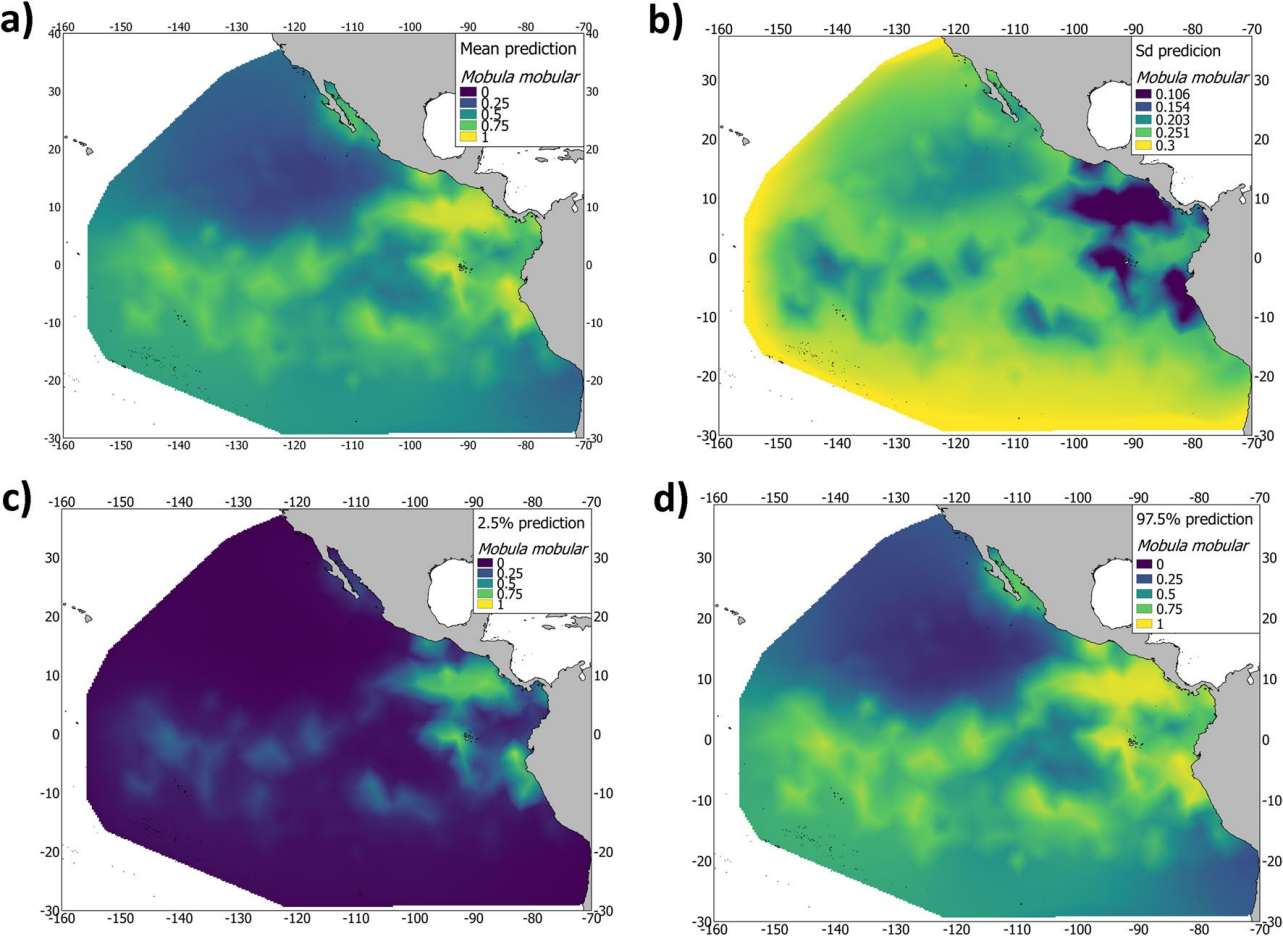
**(a)** Posterior predictive mean, (**b)** standard deviation, (**c)** 2.5% quantile and (**d)** 97.5% quantile of the presence of *Mobula mobular* bycatch from the tropical tuna purse-seine fishery (2005–2015) in the eastern Pacific Ocean. Maps were created using Quantum GIS geographic information system. Open source geospatial foundation. URL: https://qgis.osgeo.org (2014).

Prediction maps, including all the terms in the model, identified the area off the coast of Peru, the Galapagos Islands, and the Costa Rica Dome to be areas of importance for the species. With regard to the Gulf of California and the Equatorial area, both areas were properly identified by the INLA-SPDE model to be areas with high and medium probability of species presence (Fig. 2). Furthermore, the spatial effect (Fig. 1a), which indicates intrinsic spatial variability of the species distribution after excluding the environmental variables, was consistent with the probability map (Fig. 2); meaning that the variability of occurrence data for *M. mobular* could not be explained solely by the selected variables in the model, and, therefore, there is an unconsidered effect in the model.

## Discussion

This study uses a Bayesian approach to model the occurrence of *Mobula mobular* using IATTC observer bycatch data from the tropical tuna purse-seine fishery in the eastern Pacific Ocean (EPO). We consider the INLA-SPDE Bayesian approach as a complementary method to SDM traditional ones to obtain the prediction of hotspots of vulnerable species and to inform accurately management decisions.

SDMs have become one of the most powerful tools to address certain fisheries issues, such as bycatch species distribution^19^. One of the first steps to reducing bycatch mortality is to identify and manage conservation priority areas, or “hotspots”, where bycatch species may be important^19,33^. Correct identification of these areas could lead to effective spatial management strategies for their conservation. However, for regulations to be effective it requires an understanding of the spatiotemporal distribution of the species, given that wrong identification of bycatch "hotspots" can lead to erroneous mitigation practices with irreversible ecological consequences^34^. Ideally, space and time should be better incorporated into models when bycatch data is analyzed, and the choice of the best SDM model should depend on the spatial pattern of the input data^19,35^. The Bayesian approach considered in this study tried to describe these issues along with the advantages and disadvantages of using this technique in an effort to predict *M. mobular* occurrence in the EPO.

Results of the model confirm that the presence of *M. mobular* is determined by the most important seasonal upwelling systems in the EPO. The Bayesian method was able to estimate the relationship between the distribution of a species and its environment.

The non-lineal relationships observed by the models suggest that *M. mobular* may inhabit areas with different environmental characteristics but showing higher preferences for coastal, productive (with concentrations of chlorophyll between 0.1–0.2 mg·m^−3^) and low oxygen areas (around 210–220 mg/l). The presence of the species in areas with negative SSH values also suggest the association of the *M. mobular* to mesoscale process, such as eddies and coastal upwelling systems, where the food availability seems to be more abundant.

Spatial autocorrelation of residuals is normally induced by lack of a random distribution of individuals, absence of a covariate in the model or incorrect specification of the relationship between the covariate and the response variable^36^. Generally when analyzing geo-referenced by-catch data, geographic coordinates (latitude and/or longitude) are included in the models as continuous explicative variables^37,38^ given that fixed effects and, therefore, the spatial dependency of observations, is not considered. Similarly, non-random spatial variables or geographic fishing boundaries can be included as predictors in models to try to capture spatial species trends. For example, Escalle, et al.^39^ accounted for spatial autocorrelation by incorporating a contiguity matrix based on a residual’s autocovariate (RAC) as an explanatory variable in their models. However, only geo-statistical techniques intrinsically incorporate a component to account for spatial autocorrelation. Hierarchical Bayesian spatial models extend the concept of spatial autocorrelation in multilevel structures, including a spatial random effect that is a stochastic process indexed in space, which represents all spatially explicit processes that may influence the species pattern. By applying hierarchical Bayesian spatial models to species data the multiple sources of uncertainty associated with both the observed data and the species process can be included in the analysis to generate a more robust statistical inference and lead to more realistic predictions^1,35^. The standard deviation, the first and third quantile of the posterior distribution of the prediction and the spatial effect map and its uncertainty can also be mapped as another component of the model.

Moreover, one of the advantages of using INLA-SPDE approach is that is permits Delaunay triangulation over the regular grids that are normally used in SDMs. This technique congregates more information in the areas where there are more observations and, therefore, triangulation is denser in these regions and contributed to more accurate predictions. This technique is also less computationally demanding and considers the boundary effect by generating a mesh with a smooth transition from areas dominated by small triangles (which correspond to the domain of interest) to areas with larger triangles (areas out of the domain and used to avoid boundary effects). Since inference is deduced from the domain rather than the observations (which could change from year by year), the corresponding interpolation creates a better prediction surface than the traditional one using regular grid^17^. This study is also an example of these advantages. The INLA-SPDE approach was able to highlight new areas of interest, such as the Gulf of California, where the species are known to inhabit these areas. The Gulf of California is known to be an important ecological hotspot for this species^32^. Indirect exploitation of this species in the Gulf of California is mainly attributed to small-scale Mexican fisheries^40^, as there is scarce information of presence of mobulid rays due to little fishing effort of the large-scale tropical tuna purse-seine fisheries in this area^28,29^. Because this study has no access to small-scale fishery data, the correct prediction of the spatial distribution of *Mobula mobular* in the Gulf of California is even more important, as it could be considered a possible area for conservation purposes. Since most surveys and research are carried out in coastal waters (due to accessibility, funding, etc.), results from the model in this area should be taken into consideration in future analyses. The Equatorial area was also predicted to be an important area of presence for the species. In that sense, the INLA-SPDE model confirms the results obtained by Lezama-Ochoa, et al.^41^ with the correct identification of the most important areas for the species.

In this work, the model fit was different depending of the parameters considered as well as the covariates selected. For example, the inclusion of the spatial effect in the model improved significantly the model fit (lower DIC values). Therefore, we suggest including the spatial effect in future works for accounting the spatial autocorrelation of the occurrence data; really necessary to obtain real model predictions that may be used to inform management decisions. In the case of the variables chosen to explain the distribution of *M. mobular*, we also found that specific variables significantly contributed to obtain a good model fit. This is the case of “month” or “oxygen (O_2_)”. When these variables were included in the model, lower DIC, Specificity and accuracy values were obtained, representing a better model performance. These results lead to consider that the species could have a seasonal distribution and that oxygen is a limiting factor on their horizontal but also vertical distribution. However, all the covariables included in the different models were having non-linear effect on the presence of the species (since marine species do not usually respond linearly to the environment), but showing variability depending on areas or time of the day. Therefore, future work should explore a combination of linear and non-linear effects when modelling presence/absence data with environmental variables.

The spatial effect map (Fig. 1) created with the INLA-SPDE approach suggests that most of the variability in the occurrence dataset of *M. mobular* could not be explained by only the variables selected by the model. This could be true for oceanographic variables related to productivity features, such as upwelling systems, e.g., chlorophyll and sea surface height. The spatial effect represents the intrinsic spatial variability of the data after excluding the environmental variables. Therefore, when the pattern of the spatial map is similar to the map of the species prediction, it implies that there is an unconsidered effect that is driving the majority of the observed spatial distribution. In that sense, including the spatial effect as another component in the model improves model fit in addition to identifying the spatial effects that affect the distribution of the species of interest^42^.

The Bayesian approach uses probability distributions to model uncertainty in the value of parameters^43^. In that sense, not only is a point estimate of the probability of presence obtained, but it is also possible to assess the uncertainty surrounding an estimation^20^. Indeed, by using INLA-SPDE approach, it is possible to obtain the classical statistics, including standard deviation and the credible interval of the posterior probability of occurrence of the species, therefore providing an explicit quantification of the uncertainty associated to the prediction trough spatial maps. Explicitly quantifying uncertainty through spatial maps is essential to providing end-users with a reliable species distribution to determine management options.

INLA-SPDE is a relatively new approach, it is continuously being tested and improved. INLA models can also deal with traditional smoothing approaches (such as GAMs) but they also provide full inference by quantifying the uncertainty of each model parameter in a fast computational way compared to traditional MCMC simulations^17,42,44,45^. Moreover, INLA models also offer additional advantages, such as the capability to (i) simultaneously calculate inference and prediction, (ii) deal with missing data or (iii) consider data biases (e.g., survey effort can be incorporated into the models as a spatial-random effect)^10,17^.

Although the number of studies where INLA models have been compared to other approaches using fisheries data is limited^1,16,20,42^, the available studies have shown good results using Bayesian approaches. However, improvements are still needed. For instance, Lezama-Ochoa, et al.^41^ found that the frequentist GAM model is, from a computational point of view, a faster predictive technique than INLA. The model used by Lezama-Ochoa, et al.^41^ ran in a few minutes, whereas the INLA models took hours for each trial. INLA becomes quite slow when estimating non-linear posterior distributions of the covariates in a large datasets^12,46^, such as the IATTC database. When lineal components were considered, it took minutes to run models compared to approximately one hour with the non-linear relationships, however, the predictions were less precise when linear relationships were modeled (Supplementary Table S4).

The Matérn covariate function was used to model spatial autocorrelation. The correlation of every cell with every other cell in the modelling approach has a high computational cost, known as the big *n* problem^13^. The SPDE approach is normally used to address this problem, i.e., dealing with a big dataset that requires some additional computational time^12^. As such, the regression model process is faster and easier. Specific distribution models should be developed, depending of the objective of the study and the data limitations. The present study revealed that when either multiple factors or complex relationships are included in the INLA-SPDE model, the running process finished but the estimation was difficult to interpret. For example, when the variable “Type of set” (Dolphin set = 1, Floating object sets = 2, School sets = 3) was considered to be a factor (in preliminary analysis of the model) in both the estimation and the prediction, estimation of the model was correct but the evaluation and prediction was wrong.

Thus, INLA-SPDE models still face some difficulties when it comes to dealing with factors when compared to frequentist GAM models that provide easy interpretation of the ecological relationships. When “type of set” was introduced as a dummy variable in the prediction, the results improved considerably (Supplementary Table S4). This does not necessarily mean that this is the best model, but it is a good option to obtain correct predictions with our data. Regarding the standard errors or set type dummy variables (1 and 0), they seemed very large. The model without type of set showed an increment in DIC of 699.62 (Option 8, Supplementary Table S4). SD gives some rapid information about the degree of “balance” in the data from groups coded 0 and 1. For example, hypothetically the mean for the set type “Dolphin” equal to 0.95 would mean that 95% of our sample is coded 1 and the rest 0. The same in the case of “School” set type. For “Floating object”, the mean is be sensibly lower than those for “Dolphin” and “School” set type, indicating that the data are less balanced for the groups determined by the values of “Floating object”. The dummy variables included in the model, in this case the type of set, had an effect on the response variable (i.e. the distribution of the species). The negative values estimated from the model in the case of the Fishing Aggregating devices show a weak preference of these species for areas where FAD fishery is operating. This is corroborated by the fact that mobulid rays seem to be found significantly more in Dolphin and School sets compared with floating object sets^41^. The reason are unknown, but probably is due to the distribution of FAD sets in open ocean far away from coastal areas; where the productivity is much lower and, hence, mobulid rays do not find high aggregations of food available as in coastal areas. Moreover, mobulid rays do not seem to show a strong aggregating behavior around FADs as other pelagic species, such as sharks. Their preference for shallow and productive waters makes them more likely to be found in areas of the other two types of sets. This fact could explain why the variability of mobulid presence in the case of the floating object sets was so high.

Moreover, for the INLA-SPDE approach, careful consideration should be given to the selection of prior distributions or the triangulation process given that the wrong choice could lead to biased results and, therefore, more options should be compared to improve performance of the Bayesian model.

Regarding evaluation of the model predictions, there are not many differences between frequentist and Bayesian approaches. For example, Lezama-Ochoa, et al.^41^ obtained similar accuracy indices with slightly better AUC values found in the frequentist GAM model (0.92) than in our INLA model (0.88).

However, in the case of the Sensitivity index, the INLA model revealed better values (0.61) than the frequentist model (0.44). This result leads us to suggest that the prediction should be more correct in the case of the INLA-SPDE model.

In any case, as this conclusion is based on a comparison between similar models with the same environmental variables, more research is needed to compare different SDM algorithms and model parametrizations of different environmental variables. One of the objectives of this study was to explore the weaknesses and strengths of the INLA model when using observer bycatch information to model the habitat of a data poor species *Mobula mobular*. Ultimately, selection of the best model should be determined based on the objective of the study and the data. One limitation of this work arises from the lack of detailed fishing effort information. Therefore, it wasn’t possible to account for the effect of the number of sets in a particular grid on the probability of presence of the species. Future studies should consider the inclusion of fishing effort as an offset or as another explanatory variable in the model especially when modelling abundance.

From a conservation point of view, *M. mobular*, along with the rest of mobulid rays, has recently included in Appendix II of the Convention on International Trade in Endangered Species (CITES) (Appendix II) and Appendices I and II of the Convention of Migratory Species (CMS) (Appendices I & II)^47,48^. Given that the species could be exploited both as target and bycatch species^27^, it is believed that some populations could be declining in some regions^27,49^.

In the EPO, the IATTC adopted a resolution (Res. 15-04) that aims to reduce the mortality of these rays in purse seine vessels^50^. This conservation measure prohibits retaining onboard, transshipping, landing, storing, selling, or selling any part or entire carcasses of mobulid rays taken by purse seiners*.* Given this decision adopted by IATTC, the conservation of this species may be expected to improve in the region, however, for that best practices for handling and safe-release should be developed and implemented to ensure the highest post-release survival possible. From this perspective, prediction of the spatial distribution and hotspots will contribute to incorporate spatial strategies in the future as management options to reduce their mortality, while keeping an economically viable fishery.

This work implements a Bayesian GAM to investigate habitat occurrence of the Spinetail Devil Ray using data from the IATTC tropical tuna purse-seine fishery observer bycatch database. Using a novel approach and methodology it provides good model habitat occurrence predictions, which are as good as the predictions obtained with other algorithms (e.g. Random Forest, Maxent, GLM, etc.). These predictions are considered enough accurate to be included in future management plans by the tuna RFMOs. For example, model predictions from this work could be included in a new Ecological Risk Assessment approach (EASI-Fish)^51^ to study the impact of the fishery on data-poor bycatch species. This methodology could be extended to other mobulid rays or vulnerable bycatch species (i.e. sharks, turtles) and other Oceans to obtain accurate habitat occurrence predictions to inform management actions. The main achievement of this work was to provide novel and relevant information on the distribution of *M. mobular* that usually is only available from diver surveys or tagging studies limited to coastal areas.

To obtain realistic and accurate hotspots of the species, comparisons between different species distribution models (e.g., Random Forests, Maxent, Classification or Boosted Regression Trees) are needed. This would allow researchers to identify each model weaknesses and strengths to be taken into account when informing management decisions to protect the species. A community of researchers, in collaboration with the fishing industry, governments and the NGOs, that work together to implement science-based specific spatial management measures and plans depending on the areas of importance (i.e., nursery areas, reproductive, or feeding areas, etc.) or species characteristics (vulnerable, endemic, migratory, etc.) is essential for the conservation of mobulid rays.

## Conclusion

This study used a Bayesian approach to model the occurrence of *Mobula mobular* using data from the IATTC tropical tuna purse-seine fishery observer bycatch database in the EPO. The spatially-explicit Bayesian INLA-SPDE model performed well as it was able to account for the spatial autocorrelation in the data and quantify the uncertainty of parameters. Additionally, contrary to other SDM models using the same bycatch data, INLA-SPDE model correctly predicted areas of importance, such as the Gulf of California, where the presence of the species is known to occur. Although INLA-SPDE methods offer improvements to traditional models, we consider that both frequentist and Bayesian model approaches should still be combined in a complementary approach to benefit from the advantages of each method and, thus, better interpret the species distribution patterns of this vulnerable bycatch species to inform management decisions.

## Methods

### Species data

*Mobula mobular* bycatch data were collected between 2005 and 2015 by the Agreement on the International Dolphin Conservation Program (AIDCP) onboard observer program, which employs observers from both the National Observer Program and Inter-American Tropical Tuna Commission (IATTC). Data were collected in large purse seine vessels (> 363 t carrying capacity-Class 6) using three types of fishing modes or sets: tunas associated with dolphins (“Dolphin sets”), tunas associated with Floating objects [encountered (“Log sets”) or deployed by the fishers (“Fish Aggregating Devices or FAD sets”)] and unassociated schools (“School sets”). The difference between the fishing modes is the strategy used to find the school of tuna and how the set is performed: School sets are normally monospecific and schools of tuna are detected by sonar marks, jumpers or breezes in surface waters. Drifting Fish Aggregating Device sets (FADs) are done on floating objects and are used to attract tuna and other species around them. Finally, in the case of the eastern Pacific Ocean (EPO) tuna (mainly yellowfin tuna) they are frequently associated with groups of dolphins and, therefore, called Dolphin sets (Supplementary Fig. S1)^29^.

### Environmental variables

Nine oceanographic variables were extracted using *python* scripts from the European Union Copernicus Marine Environmental Monitoring Service (CMEMS) (https://marine.copernicus.eu/). For each fishing set (date and position between 2005 and 2015) the following variables were obtained at 1/4° spatial resolution: daily sea surface temperature (SST; in °C), daily sea surface height (SSH; in cm), daily salinity (Sal; in PSU), daily eddy kinetic energy derived from altimetry (Eke, in m^2^ s^−2^), daily heading and current speed derived from UV vectors (N–S^◦^ and W–E^◦^) (Heading; degrees; vel; m/s), monthly oxygen concentration (O2; mg/l), monthly Nitrate (Ni; mg/l), monthly phytoplankton (Phy; in mg·m^−3^), and monthly chlorophyll (Chl; mg·m^−3^) (Table 3).

**Table 3:** Summary of the environmental variables obtained from Copernicus Marine Environment Monitoring Service (CMEMS): variable acronym and name, unit, average value, minimum value, maximum value, and spatial and temporal resolution.

Variables acronym	Variable name	Units	Average	Min	Max	Spatial resolution	Temporal resolution
Depth	Depth	m	3732.41	6476.67	45.357	30 arc-s	**–**
Distance to the coast	Distance	Km*1000 (Euclidean distance)	8.907	0.059	23.026	5 arcmin	**–**
SST	Sea surface temperature	°C	25.276	16.69	29.636	0.25°	Daily
Sal	Salinity	psu	34.371	26.943	36.453	0.25°	Monthly
SSH	Sea Surface Height	M	0.246	− 0.001	0.627	0.25°	Daily
Chl	Chlorophyll concentration	mg m^−3^	0.217	0.024	1.83	0.25°	Monthly
Phy	Phytoplankton	mg m^−3^	1.611	0.427	15.369	0.25°	Monthly
O_2_	Oxygen concentration	mg/l	209.613	193.605	252.1	0.25°	Monthly
Ni	Nitrate	mg/l	4.785	0	20.173	0.25°	Monthly
Vel	Velocity	m/s	0.247	0.001	1.161	0.25°	Monthly
Ke	Kinetic energy	m/s	0.046	0	0.674	0.25°	Monthly
Heading	Direction of the current	Degrees	213.341	0	359.85	0.25°	Monthly
Type	Type of set (Dolphin vs. Floating object vs. School)	Considered as a factor in the estimation and as a dummy variable (0,1) in the prediction					

Two topographic covariates were also included in the models: bathymetry and distance to the coast. Both variables were obtained in raster format (ASCII format) from the Global Marine Environmental Datasets (GMED) database (https://gmed.auckland.ac.nz/download.html), and positions were matched with the positions of the fishing sets (Table 3).

To avoid correlation and collinearity between explicative variables, the Pearson’s rank correlation index and the variance inflation factor (VIF)^52^ were calculated before running the models. Specifically, correlation among variables was checked by performing a Pearson’s correlation test with the *corrplot* package in R software^53^. Red ellipses represent negative correlation and blue ellipses positive correlation. High correlation between two variables was represented in both cases by ellipses with thin thickness. Collinearity was tested by computing the generalized variance-inflation factors (GVIF), which are the corrected VIF values, by the number of degrees of freedom of a predictor variable. GVIF was assessed using the *corvif* function in R software. Pairs of variables with high correlation values (Pearson correlation r > 0.6) or high variance inflation (VIF > 5) were identified and only one was included in the modelling process (Supplementary Fig. S2)^38^.

### Modeling mobulid presence

Generalized Additive models (GAMs)^8^ are semi-parametric extensions of Generalized Linear Models (GLMs) that are able to model continuous and categorical variables, yet show non-linear responses by fitting smooth functions to predictor variables^54^.

The general structure of a GAM is as follows^5^:

$$g\left(\mu i\right)= \alpha +{f}_{1}\left({X}_{1i}\right)+{f}_{2}\left({X}_{2i}\right)+{f}_{3}\left({X}_{3i}\right)\dots .+ {f}_{n}\left({X}_{ni}\right)$$

where *g* is the link function (logit for binomial family), *µ*_*i*_ is the expected response variable (probability of bycatch in a binomial structure), *a* is the intercept, *f*_*n*_ are smooth functions (regression splines), and *X*_*n*_ are the covariates^5^.

Overall, the IATTC observer bycatch database recorded 260,002 species absences and 1270 species presences during the study period, obtained from surveys (i.e., sets with no presence of *M. mobular* recorded).

The INLA framework^14^ was implemented using the *inla* package in R software. A hierarchical Bayesian spatial GAM was implemented to model the *M. mobular* bycatch data^55^. INLA uses the Stochastic Partial Differential Equations (SPDE) approach^56^ for the spatial effect, which approximates a continuously indexed Gaussian Field (GF), where z(s) is a zero-mean Gaussian Markov Random Field (GMRF) in which the correlation between locations s_i_ and s_j,_ is Matérn. The smoothness of the field under this condition is typically denoted by the Kappa statistics index^57^. The spatial effect is a numeric vector that links each observation to a spatial location, and thus it accounts for independent region-specific noise that cannot be explained by the available covariates^20^. As recommended by Lindgren and Rue^57^, multivariate Gaussian distributions with zero means and a spatially-structured covariance matrix were assumed for the spatial component.

The response variable was modelled using the common binomial family and logit link function. All explanatory variables, except the type of set, were modeled using a second order random walk (RW2) latent model that allowed for possible non-linear relationships^12^. The variable *month* was included in the model as a cyclical effect. The type of set was considered a factor (Dolphin, Floating object, School) in the inference and a dummy variable in the prediction (i.e., 1 and 0 for each level of the factor, see Supplementary Table S4 for details). Blangiardo and Cameletti^10^ recommend that dummy variables be used to best deal with factors in INLA models.

Thus, the model can be specified as: species presence or absence at fishing location *i* (*i* = 1,…,n, n = 261,272) is given as y_i_, where y_i_ = 1 if species was present, and y_i_ = 0 if species was not present. We assumed y_i_ ~ Bernoulli(π_i_) where π_i_ is the probability of presence of *Mobula mobular* at location *i*. Then we define the model as logit(π_i_) = α_*0*_ + X_i_*β* + W_i_ where α_*0*_ is the intercept, *β* is the vector of regression parameters, X_i_ is the matrix of the explanatory covariates at location *i*, and W_i_ represents the spatially structured random effect at location _i_.

Because no prior information was available, a vague zero-mean Gaussian prior distribution with a variance of 100 was used for all the parameters involved. Posterior distributions were obtained for all the parameters that delimit the region of each posterior distribution by the 0.025 and 0.975 quantiles, where each unknown parameter is 95% likely to fall within this range of values^58^.

### Model selection

Different options were tested to obtain the best model. First, variables were included in the model without a smoothing function (i.e., linear relationship). Second, the influence of the spatial effect was explored by removing it from the model. Third, the type of set was included in the model as a dummy variable (Supplementary Table S4). Selection of the final models also occurred after carrying out a forward stepwise procedure. These options were evaluated by considering the Deviance Information Criterion (DIC)^59^. The DIC values were selected as they are the most common ones used to evaluate the performance of the models. Moreover, the Condition Predictive Ordinate (CPO) was also calculated. CPO is computed via its logarithmic score (LCPO) according to Roos and Held^60^. The CPO was used as effective index to evaluate the predictions as it is able to make an internal cross-validation taking each time just one value. Specifically, DIC measures the compromise between fit and parsimony in the model, and LCPO is a “leave one out” cross‐validation index to assess the predictive power of the model^17,18^. Lower DIC and LCPO values suggest better model performance.

### Model validation and evaluation

A cross-validation was applied with a k-fold partitioning method (with k = 5), to assess model performance^61,62^. The relationship between occurrence data and the environmental variables was modeled by using a training dataset (80% of data), and the quality of predictions was assessed using test data for validation (20% of data)^17,38,39^. Validation was repeated five times for the best model and results were averaged over the different random subsets^17^.

Models were evaluated to formally assess their overall predictability by calculating the Area Under the receiver-operating Curve (AUC), Sensitivity, Specificity and Kappa^63,64^. The AUC measures the ability of the model to correctly predict presences and absences, Sensitivity measures the percentage of presences correctly predicted, and Specificity measures the percentage of absences correctly predicted^65^. Kappa is a statistic index that corrects the overall accuracy of model predictions by the accuracy expected to occur by chance. The index ranges from 1 to + 1, where + 1 indicates perfect agreement and values of zero or less indicate a performance no better than random^65^. Model validation was performed using the *cmx* function of the *PresenceAbsence* package^66^ in R software.

### Model prediction

Prediction maps of the posterior mean, standard deviation, first and third quartile of probability of presence of *M. mobular* were obtained from the INLA model. Predictions were made using the *inla.mesh.project* and *raster* functions of the *inla* and the *raster* packages^14^ in R software. A Bayesian kriging was applied by treating the parameters as random variables in order to incorporate uncertainty into the prediction process^17^. Bayesian kriging is incorporated into the INLA approach through the SPDE module, which enables Delaunay triangulation around the presence/absence points in the sampling area (Supplementary Fig. S3)^57^. INLA perform inference and prediction simultaneously, by considering prediction locations to be points where the response is missing^15,17,20,42^. Once the prediction is generated in the selected locations, additional functions interpolate linearly to generate results for the entire study area. Model outputs were scaled from 0 to 1.

## Supplementary information

Supplementary Figure S1.

Supplementary Figure S2.

Supplementary Figure S3.

Supplementary Table S4.

## Data Availability

The datasets generated during and/or analyzed for the current study are not publicly available due to fishers’ confidentiality but are available from the IATTC’s Director under reasonable request. However, the dataset aggregated by 1 × 1º level are available at the public domain (https://www.iattc.org/publicdomaindata/iattc-catch-by-species1.htm).

## References

[1] Pennino, M. G., Vilela, R., Bellido, J. M. & Mendoza, M. Comparing methodological approaches to model occurrence patterns of marine species. in *Research Advances in Marine Resources *(Eds: Norton, K.)*. *(Nova Publisher, ISBN: 978-1-53612-177-3, 2017).

[2] Thuiller W (2003). BIOMOD-optimizing predictions of species distributions and projecting potential future shifts under global change. Global Change Biol..

[3] Phillips SJ, Anderson RP, Schapire RE (2006). Maximum entropy modeling of species geographic distributions. Ecol. Model..

[4] Booth TH, Nix HA, Busby JR, Hutchinson MF (2014). Bioclim: The first species distribution modelling package, its early applications and relevance to most current MaxEnt studies. Divers. Distrib..

[5] Guisan A, Edwards TC, Hastie T (2002). Generalized linear and generalized additive models in studies of species distributions: Setting the scene. Ecol. Model..

[6] Beaumont LJ, Hughes L, Poulsen M (2005). Predicting species distributions: Use of climatic parameters in BIOCLIM and its impact on predictions of species’ current and future distributions. Ecol. Model..

[7] Zhang W, Zhong X, Liu G (2008). Recognizing spatial distribution patterns of grassland insects: Neural network approaches. Stoch. Environ. Res. Risk Assess..

[8] Hastie, T. J. & Tibshirani, R. J. *Generalized Additive Models*. Vol. 43 (CRC Press, 1990).

[9] Martínez-Minaya, J., Cameletti, M., Conesa, D. & Pennino, M. G. Species distribution modeling: a statistical review with focus in spatio-temporal issues. in *Stochastic Environmental Research and Risk Assessment* 1–18 (2018).

[10] Blangiardo, M. & Cameletti, M. *Spatial and Spatio-Temporal Bayesian Models with R-INLA*. (Wiley, 2015).

[11] Paradinas I, Conesa D, López-Quílez A, Bellido JM (2017). Spatio-temporal model structures with shared components for semi-continuous species distribution modelling. Spatial Stat..

[12] Poggio L, Gimona A, Spezia L, Brewer MJ (2016). Bayesian spatial modelling of soil properties and their uncertainty: The example of soil organic matter in Scotland using R-INLA. Geoderma.

[13] Banerjee S, Gelfand AE, Finley AO, Sang H (2008). Gaussian predictive process models for large spatial data sets. J. R. Stat. Soc. Ser. B. (Stat. Method.).

[14] Rue H, Martino S, Chopin N (2009). Approximate Bayesian inference for latent Gaussian models by using integrated nested Laplace approximations. J. R. Stat. Soc. Ser. B. (Stat. Method.).

[15] Paradinas I (2015). Bayesian spatio-temporal approach to identifying fish nurseries by validating persistence areas. Mar. Ecol. Prog. Ser..

[16] Paradinas I (2016). Identifying the best fishing-suitable areas under the new European discard ban. ICES J. Mar. Sci..

[17] Pennino MG, Muñoz F, Conesa D, López-Quίlez A, Bellido JM (2013). Modeling sensitive elasmobranch habitats. J. Sea Res..

[18] Pennino MG (2016). Fishery-dependent and-independent data lead to consistent estimations of essential habitats. ICES J. Mar. Sci..

[19] Cosandey-Godin A, Krainski ET, Worm B, Flemming JM (2014). Applying Bayesian spatiotemporal models to fisheries bycatch in the Canadian Arctic. Can. J. Fish. Aquat. Sci..

[20] Munoz F, Pennino MG, Conesa D, López-Quílez A, Bellido JM (2013). Estimation and prediction of the spatial occurrence of fish species using Bayesian latent Gaussian models. Stoch. Environ. Res. Risk Assess..

[21] Rufener M-C, Kinas PG, Nóbrega MF, Oliveira JEL (2017). Bayesian spatial predictive models for data-poor fisheries. Ecol. Model..

[22] Quiroz ZC, Prates MO, Rue H (2015). A Bayesian approach to estimate the biomass of anchovies off the coast of Perú. Biometrics.

[23] Orue, B. *et al.* Comparing the distribution of tropical tuna associated with drifting fish aggregating devices (DFADs) resulting from catch dependent and independent data. in *Deep Sea Research Part II: Topical Studies in Oceanography* 104747 (2020).

[24] Orue, B. *et al.* Seasonal distribution of tuna and non-tuna species associated with drifting fish aggregating devices (DFADs) in the Western Indian Ocean using fishery-independent data. *Front. Mar. Sci.* (2020, in press).

[25] Alfaro-Cordova E (2017). Captures of manta and devil rays by small-scale gillnet fisheries in northern Peru. Fish. Res..

[26] Mas F, Forselledo R, Domingo A (2015). Mobulid ray by-catch in longline fisheries in the south-western Atlantic Ocean. Mar. Freshw. Res..

[27] Croll, D. A. *et al.* Vulnerabilities and fisheries impacts: the uncertain future of manta and devil rays. *Aquat. Conserv. Mar. Freshw. Ecosyst.* (2016).

[28] Lezama Ochoa, N. H., Martin, R., Marlon, V.N. Spatial and temporal distribution of mobulid ray species in the eastern Pacific Ocean ascertained from observer data from the tropical tuna purse-seine fishery. *Environ. Biol. Fish.* (2018).

[29] Hall, M. A. & Roman, M. Bycatch and non-tuna catch in the tropical tuna purse seine fisheries of the world. in *FAO Fisheries and Aquaculture Technical Paper**568 FAO, Rome*. https://www.fao.org/3/a-i2743e.pdf (2013).

[30] White WT (2017). Phylogeny of the manta and devilrays (Chondrichthyes: mobulidae), with an updated taxonomic arrangement for the family. Zool. J. Linn. Soc..

[31] Francis MP, Jones EG (2016). Movement, depth distribution and survival of spinetail devilrays (*Mobula japanica*) tagged and released from purse‐seine catches in New Zealand. Aquat. Conserv. Mar. Freshw. Ecosyst..

[32] Croll DA (2012). Movement and habitat use by the spine-tail devil ray in the Eastern Pacific Ocean. Mar. Ecol. Prog. Ser..

[33] Hazen, E.L. *et al.* A dynamic ocean management tool to reduce bycatch and support sustainable fisheries. *Sci. Adv.* **4**, eaar3001 (2018). 10.1126/sciadv.aar3001 PMC5976278 29854945

[34] Viana M, Jackson AL, Graham N, Parnell AC (2013). Disentangling spatio-temporal processes in a hierarchical system: A case study in fisheries discards. Ecography.

[35] Redding DW, Lucas TC, Blackburn TM, Jones KE (2017). Evaluating Bayesian spatial methods for modelling species distributions with clumped and restricted occurrence data. PLoS ONE.

[36] Beguin J, Martino S, Rue H, Cumming SG (2012). Hierarchical analysis of spatially autocorrelated ecological data using integrated nested Laplace approximation. Methods Ecol. Evol..

[37] Lopez, J., Alvarez‐Berastegui, D., Soto, M. & Murua, H. Using fisheries data to model the oceanic habitats of juvenile silky shark (*Carcharhinus falciformis*) in the tropical eastern Atlantic Ocean. *Biodivers. Conserv.*10.1007/s10531-020-01979-7 (2020, in press).

[38] Lezama-Ochoa, N. *et al.* Biodiversity and habitat characteristics of the by-catch assemblages in fish aggregating devices (FADs) and free school sets in the Eastern Pacific Ocean. *Front. Mar. Sci.* (2017).

[39] Escalle L (2016). Environmental factors and megafauna spatio-temporal co-occurrence with purse-seine fisheries. Fish. Oceanogr..

[40] Chong-Robles, J. *Análisis de la captura incidental de elasmobranquios en la pesquería mexicana de atún con red de cerco en el Océano Pacífico Oriental. CICESE*, MSc thesis (CICESE, Ensenada, Baja California, 2006).

[41] Lezama-Ochoa N (2019). Environmental characteristics associated with the presence of the Spinetail devil ray (*Mobula mobular*) in the eastern tropical Pacific. PLoS ONE.

[42] Pennino MG, Muñoz F, Conesa D, López-Quílez A, Bellido JM (2014). Bayesian spatio-temporal discard model in a demersal trawl fishery. J. Sea Res..

[43] Smith ANH (2016). Bayesian Modelling of Direct and Indirect Effects of Marine Reserves on Fishes: A Thesis Presented in Partial Fulfilment of the Requirements for the Degree of Doctor of Philosophy in Statistics at Massey University.

[44] Wade PR (2000). Bayesian methods in conservation biology. Conserv. Biol..

[45] Rue H (2017). Bayesian computing with INLA: A review. Annu. Rev. Stat. Appl..

[46] Huang J, Malone BP, Minasny B, McBratney AB, Triantafilis J (2017). Evaluating a Bayesian modelling approach (INLA-SPDE) for environmental mapping. Sci. Total Environ..

[47] Rohner CA (2017). Mobulid rays feed on euphausiids in the Bohol Sea. R. Soc. Open Sci..

[48] Lawson JM (2017). Sympathy for the devil: A conservation strategy for devil and manta rays. PeerJ.

[49] Couturier L (2012). Biology, ecology and conservation of the Mobulidae. J. Fish Biol..

[50] IATTC. Recommendations by the staff for conservation measures in the eastern Pacific Ocean. Document IATTC 89-04d. in *89th Meeting Guayaquil, Ecuador*. *Inter-American Tropical Tuna Commission* (2015).

[51] Griffiths SP, Kesner-Reyes K, Garilao C, Duffy LM, Román MH (2019). Ecological Assessment of the Sustainable Impacts of Fisheries (EASI-Fish): a flexible vulnerability assessment approach to quantify the cumulative impacts of fishing in data-limited settings. Mar. Ecol. Prog. Ser..

[52] Hahlbeck N (2017). Oceanographic determinants of ocean sunfish (*Mola mola*) and bluefin tuna (*Thunnus orientalis*) bycatch patterns in the California large mesh drift gillnet fishery. Fish. Res..

[53] Team, R. C. (2017).

[54] Wood, S. N. *Generalized Additive Models: An Introduction with R*. (Chapman and Hall/CRC, 2017).

[55] Diggle PJ, Tawn J, Moyeed R (1998). Model-based geostatistics. J. R. Stat. Soc. Ser. C. (Appl. Stat.).

[56] Lindgren, F., Rue, H. & Lindström, J. An explicit link between Gaussian fields and Gaussian Markov random fields: the stochastic partial differential equation approach. *J. R. Stat. Soc. Ser. B. (Stat. Method.)* **73**, 423–498 (2011).

[57] Lindgren, F. & Rue, H. Bayesian spatial modelling with R-INLA. *J. Stat. Softw.* **63** (2015).

[58] Dell'Apa A, Pennino MG, Bonzek C (2017). Modeling the habitat distribution of spiny dogfish (*Squalus acanthias*), by sex, in coastal waters of the northeastern United States. Fish. Bull..

[59] Berg A, Meyer R, Yu J (2004). Deviance information criterion for comparing stochastic volatility models. J. Bus. Econ. Stat..

[60] Roos M, Held L (2011). Sensitivity analysis in Bayesian generalized linear mixed models for binary data. Bayesian Anal..

[61] Kohavi, R. in *IJCAI* Vol. 14 1137–1145 (1995).

[62] Elith J, Leathwick JR (2009). Species distribution models: Ecological explanation and prediction across space and time. Annu. Rev. Ecol. Evol. Syst..

[63] Pearson, R. G. Species’ distribution modeling for conservation educators and practitioners. *Synth. Am. Mus. Nat. Hist.* **50** (2007).

[64] Fielding AH, Bell JF (1997). A review of methods for the assessment of prediction errors in conservation presence/absence models. Environ. Conserv..

[65] Allouche O, Tsoar A, Kadmon R (2006). Assessing the accuracy of species distribution models: prevalence, kappa and the true skill statistic (TSS). J. Appl. Ecol..

[66] Freeman, E. & Freeman, M. E. *Package ‘Presence Absence’.**R Package Version* 1 (2012).

