# Supplementary material for: Using a Bayesian modelling approach (INLA-SPDE) to predict the occurrence of the Spinetail Devil Ray (Mobular mobular)
Source: Sci Rep. 2020 Nov 2;10:18822. doi: 10.1038/s41598-020-73879-3 (PMC7606447; doi:10.1038/s41598-020-73879-3)

**Using a Bayesian modelling approach (INLA-SPDE) to predict the occurrence of the Spinetail Devil Ray (*Mobular mobular*)**

Nerea Lezama-Ochoa^1, 2*^; Maria Grazia Pennino ^3^; Martin A. Hall^2^; Jon López^2^; Hilario Murua^1, 4^

^1^ AZTI-Tecnalia, herrera kaia, portualdea z/g, 20110, Pasaia, Spain

^2^ Inter-American Tropical Tuna Commission, La Jolla, San Diego, CA, USA

^3^ Instituto Español de Oceanografía (IEO), Vigo, Spain

^4^ International Seafood Sustainability Foundation (ISSF), Washington, DC, USA.

**Supplementary Fig. S1.** Distribution of sets with presence of *Mobula mobular* for the years 2005-2015 in Dolphin sets (red triangles), Floating object sets (green diamond) and School sets (blue squares) from the tropical tuna purse-seine fishery in the eastern Pacific Ocean. Effort (represented by number of sets) for all bycatches (not only mobulids) created by using kernel density estimation (in orange) in Quantum GIS, 2014.


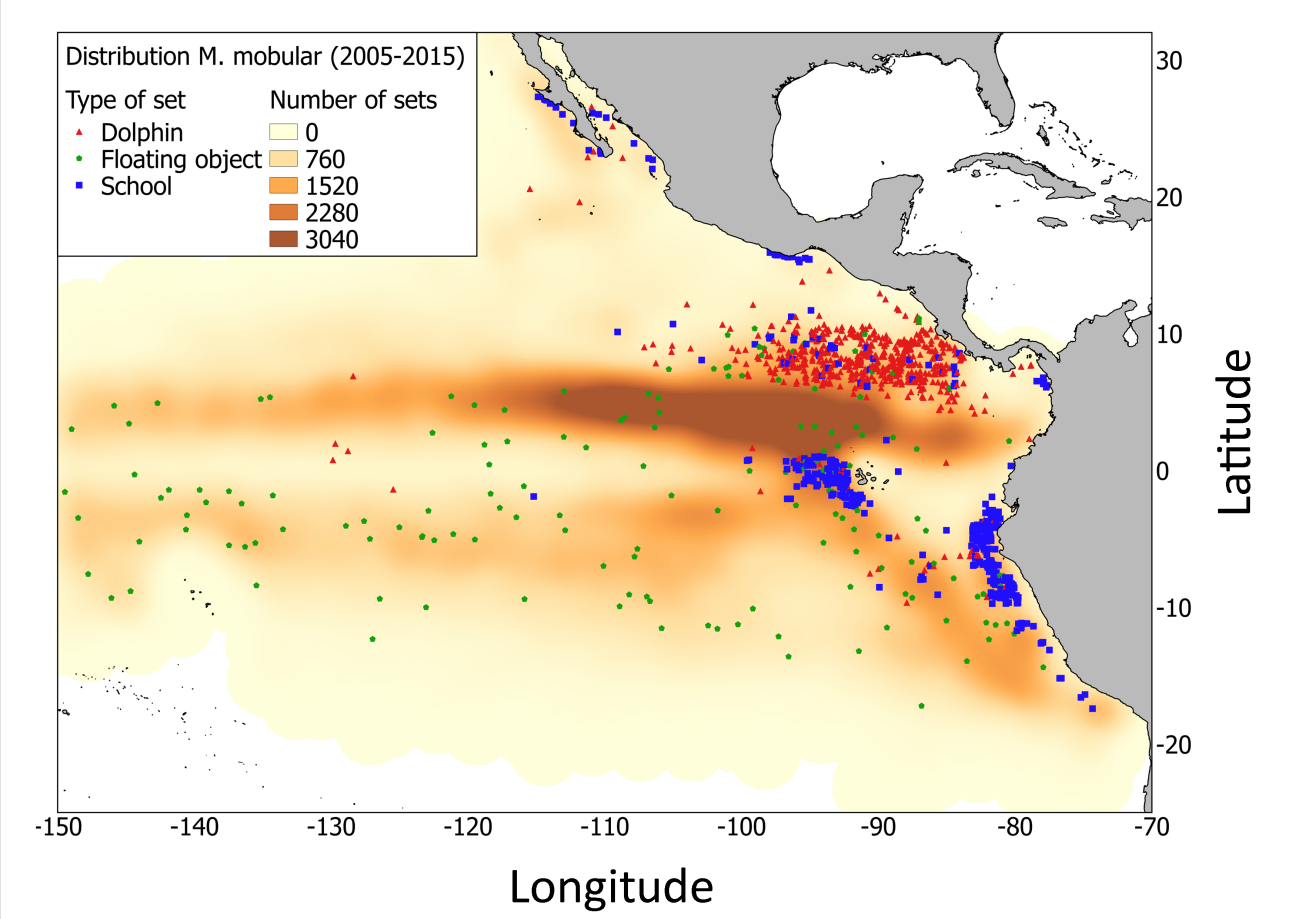

Supplement: Supplementary file 1 — Supplementary Figure S1. [file 41598_2020_73879_MOESM1_ESM.docx]
