# Supplementary material for: Using a Bayesian modelling approach (INLA-SPDE) to predict the occurrence of the Spinetail Devil Ray (Mobular mobular)
Source: Sci Rep. 2020 Nov 2;10:18822. doi: 10.1038/s41598-020-73879-3 (PMC7606447; doi:10.1038/s41598-020-73879-3)
Supplement: Supplementary file 2 — Supplementary Figure S2. [file 41598_2020_73879_MOESM2_ESM.docx]

**Using a Bayesian modelling approach (INLA-SPDE) to predict the occurrence of the Spinetail Devil Ray (*Mobular mobular*)**

Nerea Lezama-Ochoa^1, 2*^; Maria Grazia Pennino ^3^; Martin A. Hall^2^; Jon López^2^; Hilario Murua^1, 4^

^1^ AZTI-Tecnalia, herrera kaia, portualdea z/g, 20110, Pasaia, Spain

^2^ Inter-American Tropical Tuna Commission, La Jolla, San Diego, CA, USA

^3^ Instituto Español de Oceanografía (IEO), Vigo, Spain

^4^ International Seafood Sustainability Foundation (ISSF), Washington, DC, USA.

**Supplementary Fig. S2.** Study of correlation and collinearity between variables by calculating Pearson’s rank correlation and the Variance Inflation Factor (VIF). Correlation between variables was represented by red ellipses (negative correlation) and blue ellipses (positive correlation). When two variables were shown to be highly correlated the ellipse thickness was thin.


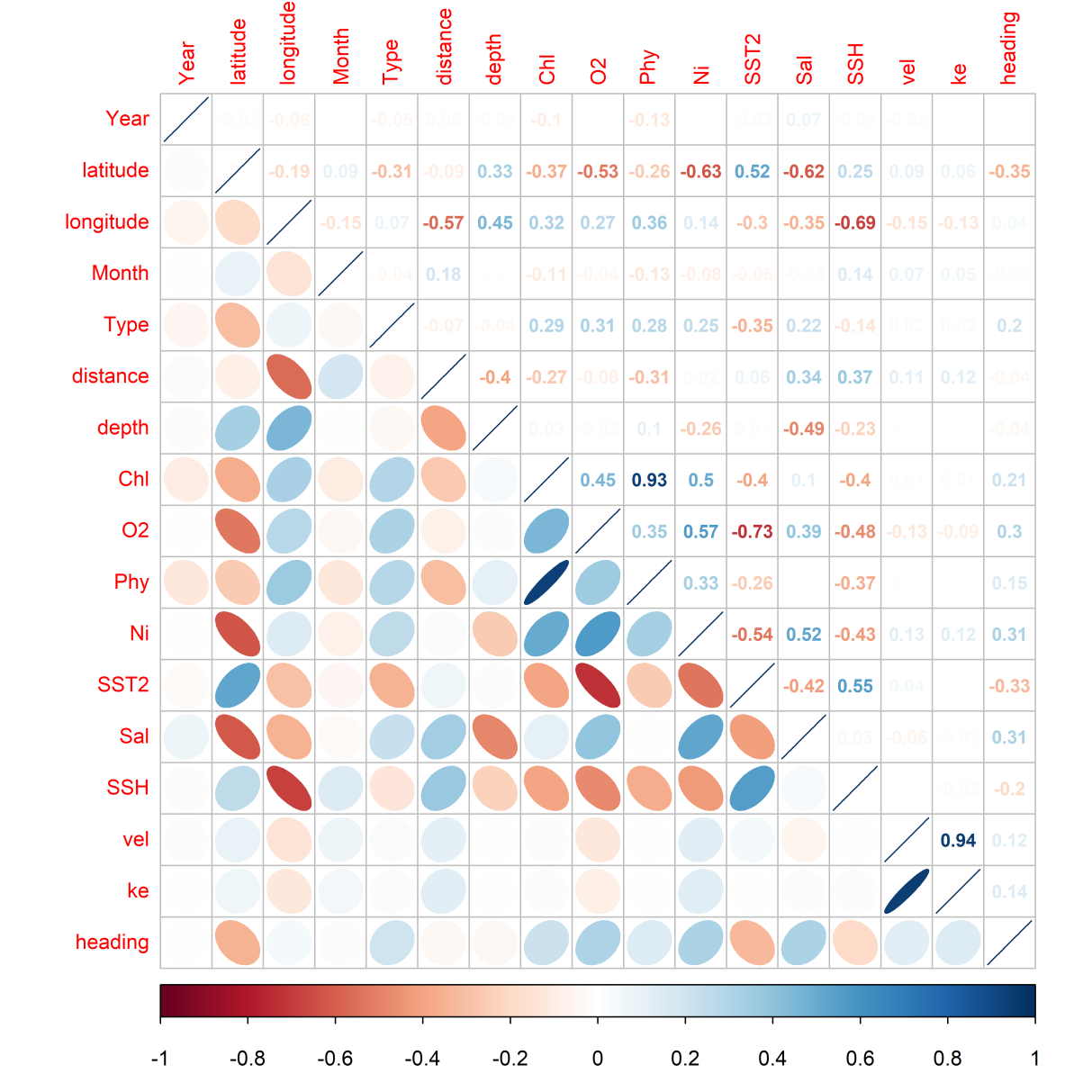


| **Variable** | **GVIF** |
| --- | --- |
| Latitude | 3.3090 |
| Longitude | 4.6796 |
| Distance | 1.7505 |
| Depth | 1.7555 |
| Type | 1.2797 |
| Year | 1.0443 |
| Month | 1.4874 |
| SST | 3.8281 |
| Chl | 13.7682 |
| Phy | 11.8841 |
| Ni | 3.2042 |
| O2 | 2.6132 |
| Salinity | 3.7194 |
| SSH | 3.2852 |
| vel | 10.0074 |
| ke | 9.4751 |
| heading | 1.2793 |
