# Supplementary material for: Using a Bayesian modelling approach (INLA-SPDE) to predict the occurrence of the Spinetail Devil Ray (Mobular mobular)
Source: Sci Rep. 2020 Nov 2;10:18822. doi: 10.1038/s41598-020-73879-3 (PMC7606447; doi:10.1038/s41598-020-73879-3)

**Using a Bayesian modelling approach (INLA-SPDE) to predict the occurrence of the Spinetail Devil Ray (*Mobular mobular*)**

Nerea Lezama-Ochoa^1, 2*^; Maria Grazia Pennino ^3^; Martin A. Hall^2^; Jon López^2^; Hilario Murua^1, 4^

^1^ AZTI-Tecnalia, herrera kaia, portualdea z/g, 20110, Pasaia, Spain

^2^ Inter-American Tropical Tuna Commission, La Jolla, San Diego, CA, USA

^3^ Instituto Español de Oceanografía (IEO), Vigo, Spain

^4^ International Seafood Sustainability Foundation (ISSF), Washington, DC, USA.

**Supplementary Fig. S3.** The Delaunay triangulation of the eastern Pacific Ocean. The red points are marking the fishing locations.


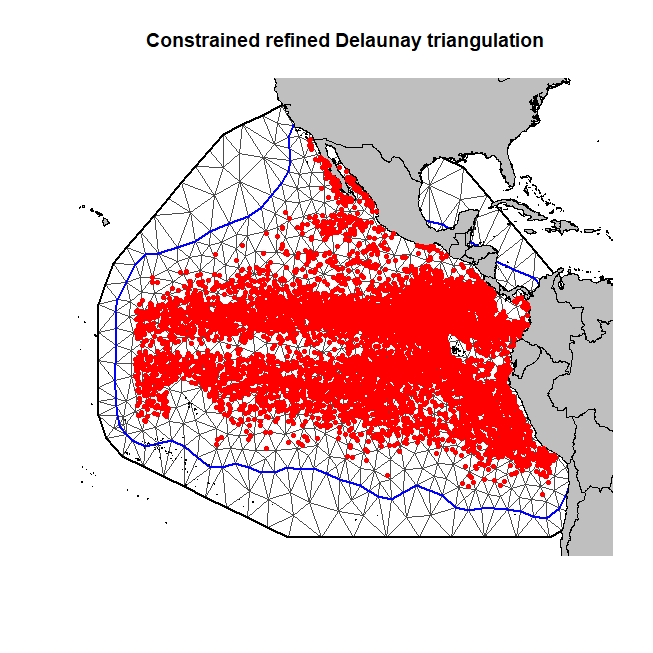

Supplement: Supplementary file 3 — Supplementary Figure S3. [file 41598_2020_73879_MOESM3_ESM.docx]
