# Supplementary material for: Using a Bayesian modelling approach (INLA-SPDE) to predict the occurrence of the Spinetail Devil Ray (Mobular mobular)
Source: Sci Rep. 2020 Nov 2;10:18822. doi: 10.1038/s41598-020-73879-3 (PMC7606447; doi:10.1038/s41598-020-73879-3)
Supplement: Supplementary file 4 — Supplementary Table S4. [file 41598_2020_73879_MOESM4_ESM.docx]

**Using a Bayesian modelling approach (INLA-SPDE) to predict the occurrence of the Spinetail Devil Ray (*Mobular mobular*)**

Nerea Lezama-Ochoa^1, 2*^; Maria Grazia Pennino ^3^; Martin A. Hall^2^; Jon López^2^; Hilario Murua^1, 4^

^1^ AZTI-Tecnalia, herrera kaia, portualdea z/g, 20110, Pasaia, Spain

^2^ Inter-American Tropical Tuna Commission, La Jolla, San Diego, CA, USA

^3^ Instituto Español de Oceanografía (IEO), Vigo, Spain

^4^ International Seafood Sustainability Foundation (ISSF), Washington, DC, USA.

**Supplementary Table S4**. Model comparision of the INLA fitted models using lineal and no-lineal relationships. Comparision criteria are: DIC= Deviance Information Criterion; LCPO= Condition Predictive Ordinate; AUC=Area Under the Curve; Sensitivity; Specificity. Variables acronyms are: Type=Type of set; Ch=Chlorophyll; SSH=Sea Surface Height; Ni=Nitrate; O2=Oxygen; Vel=current speed; Ke=eddy kinetic energy; Φ=Spatial effect.

| **Model** | **Type** | **Variables** | **DIC** | **CPO** | **AUC** | **Sensitivity** | **Specificity** |
| --- | --- | --- | --- | --- | --- | --- | --- |
| Option 1 | lineal | Year + Type + Chl + Ni + Vel | 192025.93 | 5.07 | 0.60 | 1 | 0 |
| Option 2 | lineal | Year + Type + Chl + Ni + Vel + Heading + Distance | 192055.03 | 5.07 | 0.83 | 0 | 0.60 |
| Option 3 | lineal | Year + Type + Chl + Ni + ke + Depth | 192109.50 | 5.07 | 0.65 | 0 | 1 |
| Option 4 | lineal | Type + Chl + Ni + O2 + Month | 10131.50 | 3.71 | 0.50 | 1 | 0.17 |
| Option 5 | lineal | Type + Chl + Ni + O2 + Month + SSH + Heading+ Vel+Distance | 9921.65 | 3.70 | 0.93 | 1 | 0.04 |
| Option 6 | lineal | Chl + Month + Φ | 9616.43 | 3.69 | 0.96 | 1 | 0.95 |
| Option 7 | no-lineal | Chl + Ni + SSH + O2 + Month + Type | 9339.16 | 3.31 | 0.68 | 1 | 0.09 |
| Option 8 | no-lineal | Chl + Ni + SSH + Month + Φ | 9473.30 | 3.71 | 0.93 | 0.50 | 0.97 |
| Option 9 | no-lineal | Chl+Ni+O2+ Month + Φ | 8852.79 | 3.67 | 0.70 | 0.50 | 0.96 |
| **Option 10*** | **no-lineal** | **Chl + Ni + SSH + O2 + Month + Type +** Φ | **8773.68** | 3.66 | **0.88** | **0.61** | **0.88** |

*Type of set as a factor (Dophin, Floating, School) in the estimation and as a dummy variable in the prediction.
